# Exploring dielectric and AC conduction characteristics in elemental selenium glass modified with silver halides

**DOI:** 10.1039/d4ra02999b

**Published:** 2024-07-02

**Authors:** Anil Kumar, Vishnu Saraswat, A. Dahshan, H. I. Elsaeedy, Neeraj Mehta

**Affiliations:** a Department of Physics, Banaras Hindu University Varanasi 221005 India dr_neeraj_mehta@yahoo.ac.in; b Department of Physics, College of Science, King Khalid University Abha 61413 Saudi Arabia

## Abstract

In this research work, we have examined the influence of silver halide doping on the dielectric dispersion and AC conduction of elemental selenium. The in-depth investigation shows that when the dopant silver halides are incorporated, there are noticeable changes in the parent selenium's dielectric constant (*ε*′), dielectric loss (*ε*′′), and AC conductivity (*σ*_ac_). When we frame the discussion of the obtained results with the relevant transport models, we found that in pure selenium and Se_95_(AgI)_5_, conduction is primarily due to polaron hopping and follows the correlated barrier hopping (CBH) model. In contrast, Se_95_(AgBr)_5_ predominantly exhibits non-overlapping small polaron tunneling (NSPT). Interestingly, Se_95_(AgCl)_5_ demonstrates both NSPT and CBH conduction mechanisms, depending on the temperature range: NSPT is dominant between 303 K and 313 K, while CBH prevails from 318 K to 338 K. Additionally, our findings revealed the presence of both the Meyer–Neldel rule (MNR) and its reverse in the prepared silver halide chalcogenide alloys. The best optimization of dielectric constant and loss is observed for silver iodide as compared to silver chloride and silver bromide. Comparison with other silver-containing chalcogenide glasses indicates the better dielectric performance of the present silver halides containing selenium.

## Abstract 2

Schematic illustration of the steps involved in the synthesis route of the present samples by doping of silver halides in glassy Selenium.

## Graphical abstract

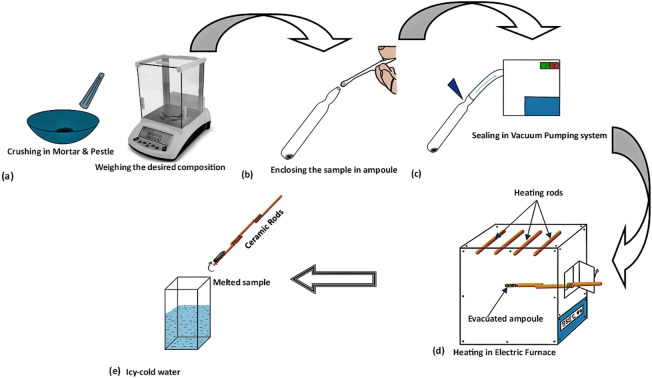

## 1. Introduction

Glasses and ceramics have emerged as pioneering subjects in advanced materials research, offering innovative solutions to contemporary global challenges across diverse domains such as energy, the environment, and information/communication technology. Their special qualities and broad range of uses drive technical progress and encourage long-term improvements in everything from optics and electronics to infrastructure and healthcare. Chalcogenide glasses (ChGs) have garnered significant attention due to their distinctive properties and capabilities, making them highly promising for applications in photonics and optoelectronics. ChGs are non-crystalline substances composed of chalcogen atoms (typically S, Se, or Te) and one or more electropositive atoms from the p-block of the periodic table.^1,2^ Ag-doped chalcogenides, in particular, are of considerable interest with a wide range of applications, and the understanding of their properties is rapidly advancing.^3^

The slight disparity in electronegativity among the constituents makes ChGs predominantly covalently bonded, which results in a bonding strength lower than that of oxide materials. Consequently, glass formation is possible over a wide range of compositions, allowing for the creation of both homonuclear and heteronuclear connections and producing remarkable stability in non-stoichiometric molecules. This structural adaptability and diverse bonding nature enable unparalleled capacity for doping and alloying, facilitating precise manipulation of the optical and electrical properties of these materials. This versatility not only promotes groundbreaking advancements in optoelectronics but also holds potential for novel applications in emerging technologies such as flexible electronics, photovoltaics, and advanced sensing devices.^4^

Adding specific metallic modifiers as foreign elements to chalcogenide glasses provides valuable information on the temperature-dependent dielectric and AC conduction response at various audio frequencies, correlating the electrical properties with structural modifications.^5–7^ Dielectric relaxation is used to study dipole behavior in materials as a consequence of varying temperatures and frequencies, aiding in understanding the dielectric response. Examining the structure of chalcogenide glasses is crucial for gaining deeper insight into transport mechanisms. AC conductivity measurements are widely used to investigate defect centers in disordered systems, based on the assumption that these centers are responsible for this type of conduction. By examining a material's dielectric response, one can learn more about the dynamics of charge carriers and the underlying conduction mechanisms. For example, dielectric relaxation studies can provide insights into the presence and dynamics of localized centers and the frequency and temperature dependence of the dielectric constant and conductivity.^8^ The study of thermally triggered AC conduction as a function of temperature and frequency offers insights into the electrical relaxation of dielectric materials.^9^ When silver halides are added as a dopant, the electrical characteristics of selenium are improved, making them very well suited for use as nonlinear optical materials. This improvement has great potential for use in different applications, including actuators, sensors, and capacitors in solid-state electronic devices. Ferau *et al.*^10^ synthesized composites of silicate bioactive glasses and silver iodide (AgI) and reported their possible tissue engineering applications. Sayyed *et al.*^11^ observed that the radiation shielding effectiveness of borate glasses embedded with low amounts of CrO_3_, and high levels of Na_2_O can be improved by doping AgI. Marasanov *et al.*^12^ reported the formation of the Ag–AgBr nanostructures by using the low-temperature ion exchange method and followed heat treatment in bromide sodium–zinc–aluminosilicate glass. They observed that the presence of a photocatalyst with Ag–AgBr nanostructures in the surface layer of glass under ultraviolet irradiation leads to better photocatalytic properties and the samples can be applied in devices for the photocatalytic decomposition of water to produce hydrogen. Bokova *et al.*^13^ developed the potentiometric chemical sensors for sodium detection by doping metal halides in (Ga_2_S_3_)_26_(GeS_2_)_44_ chalcogenide glasses.

In present studies, we have doped silver halides in the elemental selenium glass instead of alloying silver with selenium or selenium-rich ChGs since the alloying of silver with chalcogens has already been performed by various research groups in the past.^3^ This paper unveils the outcomes of dielectric dispersion and AC conduction in Se_(100−*y*)_(AgX)_*y*_ glass ceramics (X = Cl, Br, and I; *y* = 0 and 5 weight percentages).

## 2. Material preparation

**Fig. 1:**
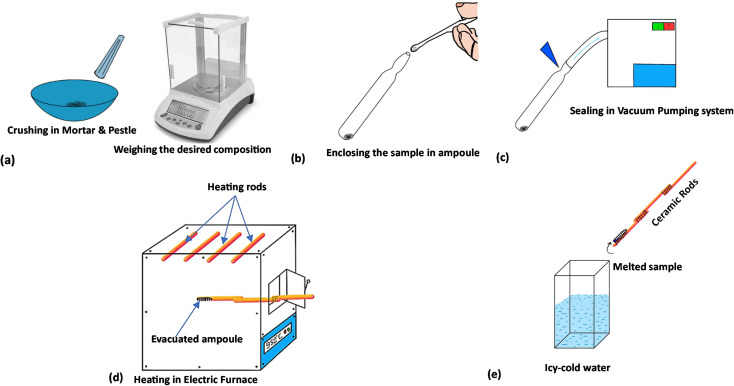
Illustration of different steps involved in the melt-quench synthesis route: (a) crushing of the samples in powdered form for accurate weighing, (b) enclosing the desired compositions in quartz tubes known as ampoules, (c) sealing of the ampoules using high-vacuum pumping system, (d) rocking and melting of the samples during the high-temperature processing inside an electric furnace, and (e) rapid cooling of the tubes inside a tank of chilled water.

Materials Se_100_(AgX)_0_, Se_95_(AgCl)_5_, Se_95_(AgBr)_5_ and Se_95_(AgI)_5_ were produced using the conventional melt quench method. It has been reported that ionic conduction becomes dominant if we add the high silver and silver halide content in chalcogenides glasses.^3,14^ Thus, we have used 5 weight percentages of silver halides to maintain the electronic conduction in the obtained samples. In the first step, the elemental selenium and powdered silver halides (AgCl, AgBr, and AgI) were precisely weighed to synthesize the present samples. We have procured the 5 N pure selenium and 4 N pure silver halides from Thermo Fisher, USA to ensure the purity of the prepared samples. The materials were sealed inside the quartz ampoules by using a high vacuum pumping system (steps two and three), followed by continuous heating for ten hours in an electric furnace at 800 °C (step four). Eventually, the ampoules were taken out of the furnace and swiftly dipped into ice-cold water to cool the molten substance (step five), and the quartz ampoules were broken carefully to release the prepared bulk materials. Fig. 1 shows a pictorial representation of all the steps described above.

## 3. Experimental basis

An X-ray diffractometer was used to record the XRD pattern of each produced sample.

**Fig. 2:**
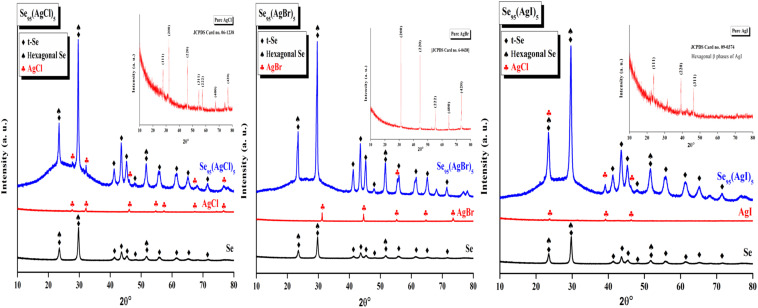
XRD patterns of the present samples (shown by blue color). XRD patterns shown by black and red color belong to elemental Selenium and Silver halides. Insets show the corresponding JCPDC cards of silver halide used in the present study.

The XRD pattern of all the prepared samples was recorded by an X-ray diffractometer (Cu-K_α_ radiation *λ* = 1.54 Å). The tube was maintained at 45 kV and 40 mA. The XRD pattern of all Se_95_(AgX)_5_ samples is shown in Fig. 2 (blue) in comparison with parent selenium (black) and dopant silver halide salt (red). Only a few peaks of insignificant intensities were observed over the broad hump *i.e.*, the first sharp diffraction peak (FSDP), which is indicative of intermediate-range ordering in the current samples, was one of only a few faintly visible peaks over the broad humps However, we have identified some distinguished peaks that can be indexed. XRD patterns indicate the presence of crystallinity, overlaying the amorphous glassy matrix. The materials having this kind of crystallinity are known as glass ceramics. Peaks are identified using literature review, X'Pert Highscore Plus software, and matched with the JCPDS database. The primary most intense peaks of crystalline t-Se *i.e.*, trigonal Se (COD 9008579) are visible at 2*θ* values of 23.5°, 29.7°, and 51.7°, which correspond to the crystal planes (100), (101), and (201), respectively.^15,16^ Also, the lattice index obtained was close and consistent with standard data for hexagonal selenium (JCPDS 96-901-2502). For Selenium, recorded reflection peaks are 23.55°, 29.69°, 41.32°, 43.65°, 45.36°, 48°, 51.7°, 56.11°, 61.67°, 65.23° and 71.7° which correspond to the crystal planes (100), (101), (110), (102̄), (111), (200), (201), (112), (202̄), (210) and (113), respectively.^17^ Peaks of different silver halide salts are identified and presented in the inset of the XRD patterns. AgCl nanocrystal diffraction peaks at 27.7°, 32.2°, 46.18°, 67.3°, and 76.78° could be correlated with the (111), (200), (220), (400), and (420) planes of the cubic-phase AgCl. In the case of Se_95_(AgBr)_5_, AgBr nanocrystal the only diffraction peaks at 55.3° identified which could be correlated with the (222) plane of the cubic-phase AgBr and all other phases of AgBr are missing. AgI nanocrystal diffraction peaks at 23.8°, 39.2°, and 46.3° could be correlated with the (111), (220), and (311) planes of the cubic-phase AgI.

**Fig. 3:**
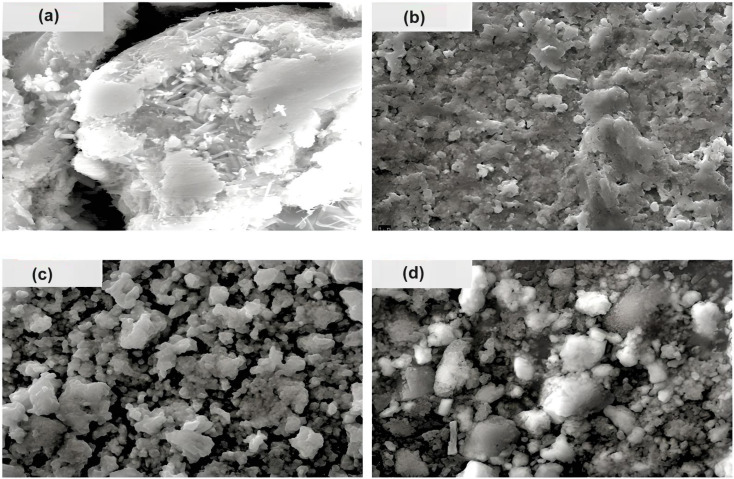
SEM images of the present samples: (a) Se, (b) Se_95_(AgCl)_5_, (c) Se_95_(AgBr)_5_, and (d) Se_95_(AgI)_5_.

**Fig. 4:**
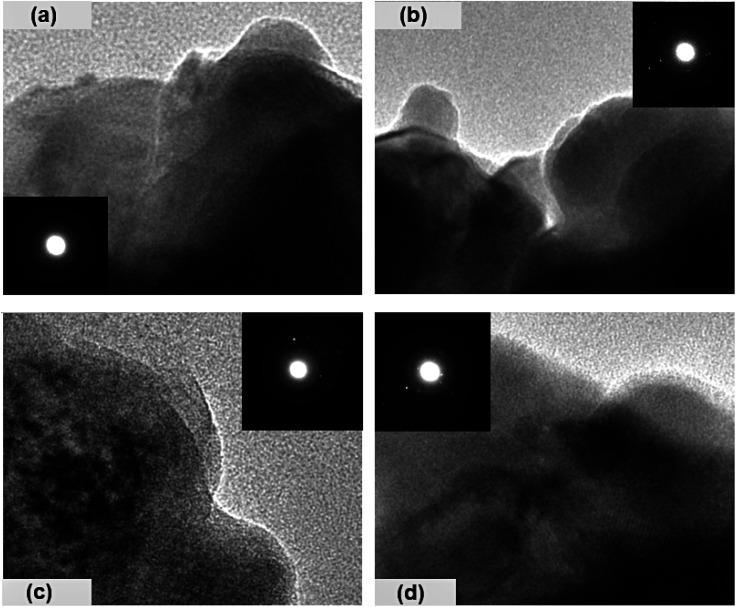
TEM images of the present samples: (a) Se, (b) Se_95_(AgCl)_5_, (c) Se_95_(AgBr)_5_, and (d) Se_95_(AgI)_5_.

The surface morphology of the prepared chalcogenide system is analyzed using a scanning electron microscope (SEM), as shown in Fig. 3 for the present samples. The whole surface of each sample has a non-uniform and irregular microstructure, as seen in the SEM image. However, when silver halides are incorporated into the parent selenium sample, the microstructural morphology changes. Now, the particles are aggregated with a more granular morphology that seems to be tightly packed with irregular shapes. Thus, we observe small, irregular, tightly packed granular particles aggregated in the selenium matrix after the incorporation of AgX. The TEM images of the present samples are depicted in Fig. 4. The particles exhibit a heterogeneous size distribution, with various lateral dimensions formed by stacked layers, indicative of microparticle clustering and the consolidation of these clusters within the glass matrix. Some layers have undergone exfoliation, leading to more refined edges, likely due to the formation of a passivation layer on the surface. Consequently, all samples display microstructural characteristics akin to materials with a glassy nature. However, a comparison of the electron diffraction patterns (EDP) between pristine selenium and silver halide-doped selenium samples reveals that the pristine selenium sample exhibits a single diffuse ring, while the Se-AgX samples display intermittent rings corresponding to indistinct reflections, signifying medium-range ordering (MRO). In chalcogenide glasses (ChGs), metastable clusters with extensive surfaces can be considered as undersized clusters with an internally ordered structure. The ChGs consist of perceived clusters that serve as intermediate-range ordered microstructural units of diverse geometries (*i.e.*, each unit has a unique size and structure). Thus, these glasses may contain various configurational units. The intermediate-range ordered structure of glasses resembles that of fractional segments of chain-layered crystals. The glass network of Se_95_(AgX)_5_ glass-ceramics comprises a substantial fraction of Se–Se homopolar bonds and a small yet significant fraction of silver-halide salts. Consequently, the appearance of discontinuous rings in the electron diffraction pattern of the silver halide-doped sample can be attributed to the MRO.

The aforementioned analysis, based on X-ray Diffraction (XRD), Scanning Electron Microscopy (SEM), and Transmission Electron Microscopy (TEM) characterization, elucidates the existence of crystallinity superimposed on an amorphous glassy matrix, thereby indicating the formation of glass-ceramics. When silver is incorporated into chalcogenide glasses (ChGs), it acts as a bridging atom among individual selenium chains, facilitating the formation of a network structure that ultimately leads to the development of a glassy material. Conversely, doping with silver halides may result in the shortening of selenium chains, as the monovalent halide ions can attach to the terminal points of the chains or interact with lone-pair electrons of selenium atoms within the chains. Thus, in contrast to silver-doped ChGs, silver halide doping does not lead to the formation of glass with short-range ordering but rather promotes the development of glass ceramics.

**Fig. 5:**
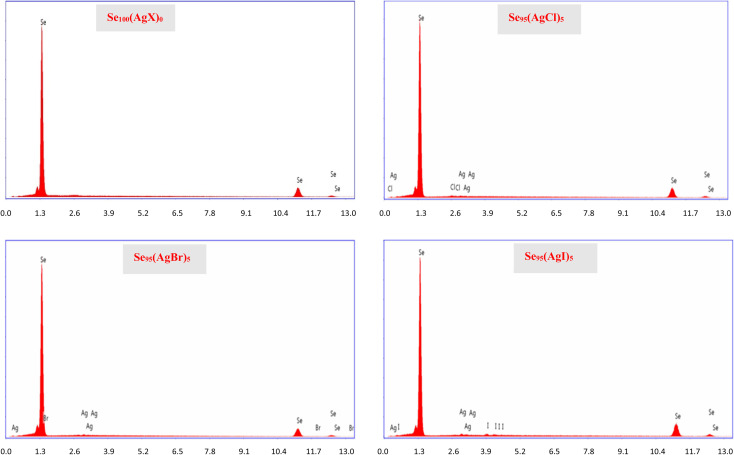
EDX spectra of the present samples.

To ascertain the precise composition of the constituents within the samples, we employed energy-dispersive X-ray spectroscopy (EDX). Fig. 5 shows the EDX spectra of the present samples. In our examination of the EDX pattern, we did not find any discernible compositional variation. This became possible because of careful weighing in a sensitive weighing balance (Contech Electronic Balance, India; model: CA-44) of sensitivity 0.1 mg.

**Fig. 6:**
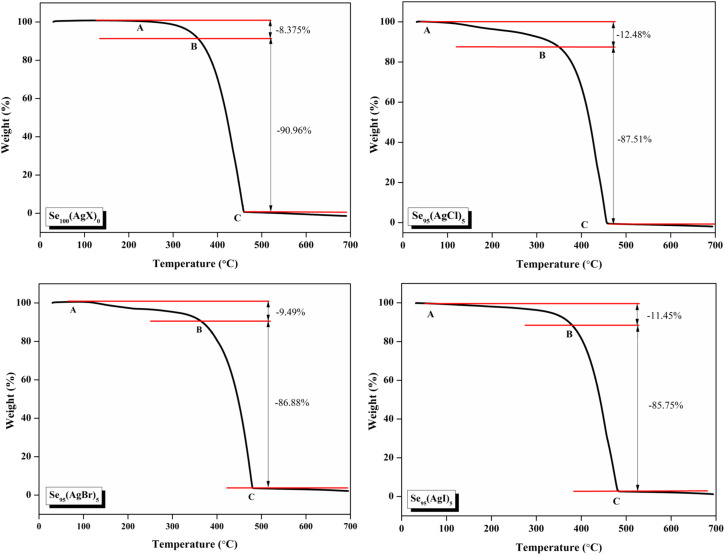
TGA scans of the present samples.

The thermal stability of these samples was investigated by Thermogravimetric analysis (TGA). A PerkinElmer thermal analyzer is used to obtain the TGA thermographs. The nitrogen gas was continuously flowed at a rate of 20 mL min^−1^, and the temperature was increased at a rate of 10 °C min^−1^ from 30 °C to 700 °C. TGA thermograms of the present samples are shown in Fig. 6. The sample exhibits a comparable two-stage deterioration, as shown by this figure, with a slow mass loss from about 100 °C to 380 °C, or about 10% of its initial weight lost, and a significantly quicker mass loss (about 85–89% of its initial weight lost) between 380 °C and 460 °C. These results show that the prepared samples have good thermal stability nearly up to 400 °C.

Using a digital LCR meter (Wayne Kerr Electronics, Model: 4100), we performed electrical measurements to examine the temperature/frequency dependence of *ε*′, *ε*′′, and *σ*_ac_. The loss tangent (tan(*δ*)), and capacitance (*C*) were measured directly in the frequency span of 0.5 kHz–500 kHz.

## 4. Theoretical basis

By considering only the existence of bound charges in chalcogenide polymers, we can use the relation *σ* = *ωε*_0_*ε*′′ to estimate the AC conductivity directly. Guintini and his fellow researchers developed a practical method for examining dielectric dispersion by evaluating variations in dielectric loss (*ε*′′) across a specified temperature (*T*) or frequency range.^18^1
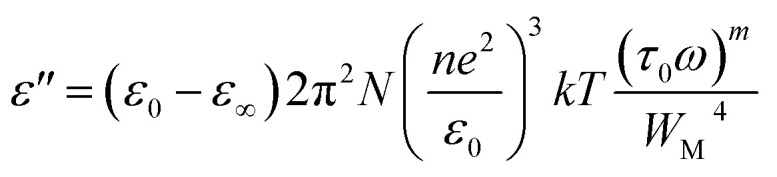
where *ε*_0_ is the free space permittivity, *ε*_∞_is the dielectric constant at extreme frequencies, *N* is the concentration of localized (defect) states, *n* is the number of hopping electrons, *ω* is the angular frequency (*ω* = 2π*f*), and *W*_M_ is the energy required to move the electron from one site to the infinite, and other symbols have their usual meaning. The above equation can also be written as:

2 *ε*′′ = *Bω*^*m*^

The constant *B* denoted in the above equation is represented by the following expression:3
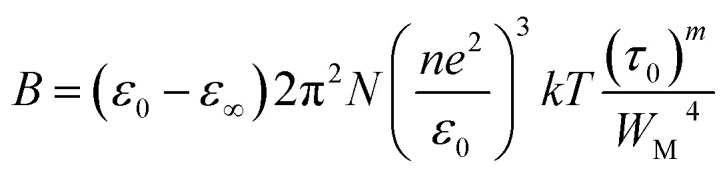
along with a frequency exponent *m*, characterized by its negative values, and it is formulated as follows:4
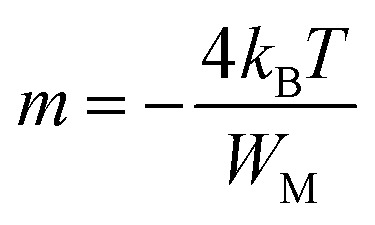


Many scientific theories have been proposed by researchers to establish a connection between the conduction mechanism of AC conductivity and the behavior of *s*(*T*) in amorphous (glassy) materials, which arises from electron hopping or tunneling processes. These theories encompass the following models: (i) CBH, (ii) NSPT, (iii) overlapping large polaron tunneling (OLPT) model, and (iv) the Quantum-Mechanical Tunnelling model (QMT). The central objective of these models is to provide insights into the underlying mechanism governing alternating current (AC) conduction.^19^ The CBH model demonstrates a decrease in the exponent *s* as temperature increases.^20^ In contrast, the NSPT model shows an increase in the exponent *s* with rising temperature.^21^ Upon an increase in temperature, the OLPT model indicates a decline in the parameter *s*, which attains a minimum at a specific temperature before exhibiting an upward trend once more.^22^ Conversely, the QMT model predicts that the exponent *s* remains largely invariant with temperature or exhibits a slight increment, maintaining a fixed value of 0.8.^23^

The electrical conductivity of chalcogenide samples, as indicated in ref. 24, is characterized by alternating current that varies with frequency due to the out-of-phase movement of charges “Universal dielectric response (UDR)”:

5 *σ*_ac_(*ω*) = *Aω*^*S*^

The power law exponent “*S*” measures electrical conduction and relaxation behavior and it shows how strongly polarons and charge carriers interact with their environment. Importantly, *s* is also observed to be temperature-dependent.

According to the CBH model, conduction is affected by the motion of electrons that get stuck within two defect centers and pass across an isolated Coulomb barrier. This model, introduced by Pike^20^ in 1972 for the translocation of a single electron, was later expanded by Elliot in 1977 to accommodate the synchronized hopping of two electrons.^25^ Because of Coulomb interactions, the elevation of the barrier depends on the distance between the two defect states. The potential wells overlap and the barrier height from *W*_M_ to *W* decreases as the locations grow closer together (a decrease in separation indicated by *R*_*ω*_), as described by the equation:6
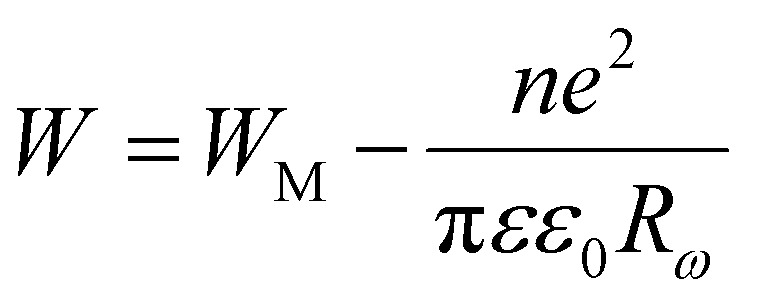


According to the CBH model, exponent *s* can be expressed as:7
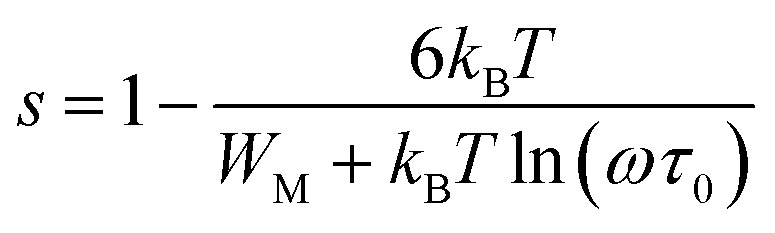


The AC conductivity is expressed as follows:8
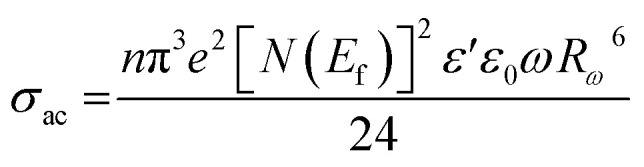


The polaron hopping distance *R*_*ω*_ is given by the expression:9
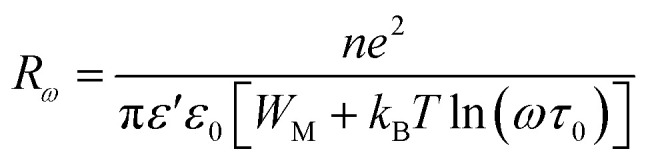


According to the NSPT model, exponent *s* can be expressed as:10
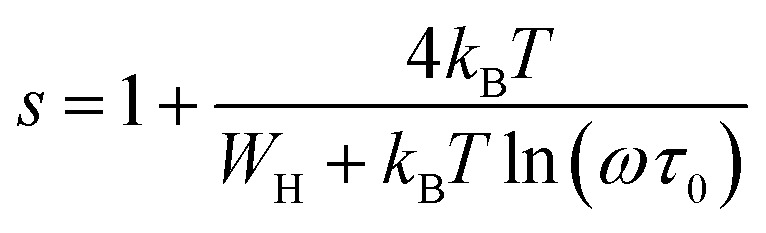


The AC conductivity in terms of the density of localized states *N*(*E*_f_) is expressed as follows:11
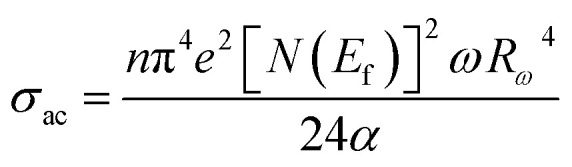


In eqn (11), α^−1^ denotes the localization length of the wave function of small polaron.^26,27^

The *R*_*ω*_ in terms of *α* can be expressed as:12
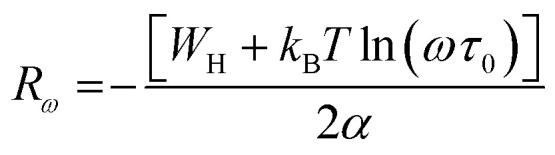


## 5. Result and discussion

The dielectric constant, which can be computed by dividing the material's capacitance by the vacuum's capacitance, indicates a material's capacity for conserving electrical energy.^28^ We have calculated the dielectric constant from the measured value of capacitance *C* in the following relation:13
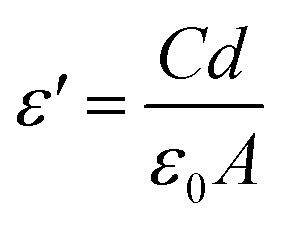
Here, *d* and *A* are the respective thickness and cross-sectional area of the pellet-shaped sample while *ε*_0_ is the well-known permittivity of free space.^28,29^

Additionally, dielectric loss *ε*′′ is calculated by the relation:

14 *ε*′′ = *ε*′ tan *δ*

**Fig. 7:**
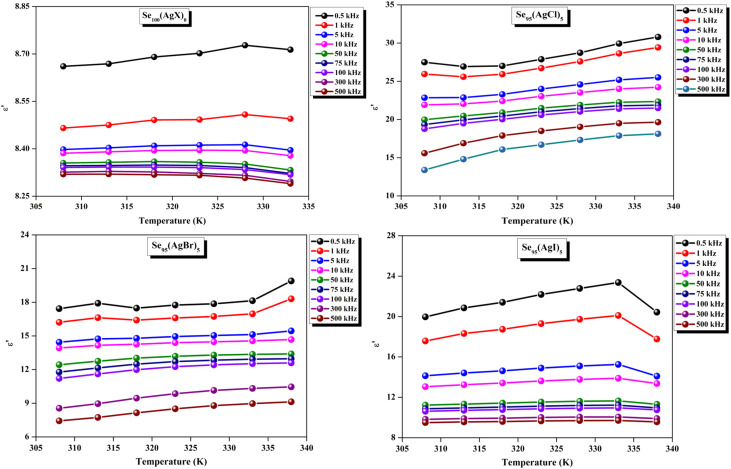
Temperature dependence of dielectric constant for the present samples.

**Fig. 8:**
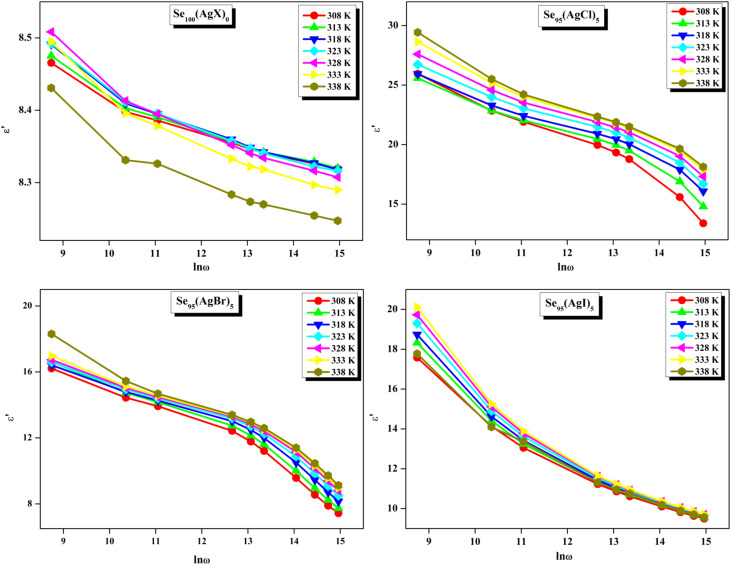
Frequency dependence of dielectric constant for the present samples.

**Fig. 9:**
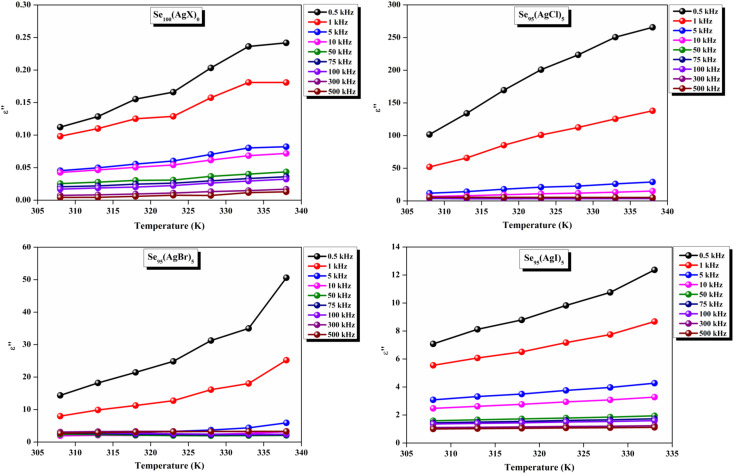
Temperature dependence of dielectric loss for the present samples.

The loss tangent, which is symbolized as tan *δ*, plays a role in computing the dielectric constant. In this context, *ε*′ stands for the real component of the dielectric constant. The variation of *ε*′ with temperature for prepared elemental glassy Selenium and multicomponent Se_95_(AgX)_5_ (X = Cl, Br, and I) chalcogenide glasses are depicted in Fig. 7. This shows that the *ε*′ increases with the applied temperature in the studied frequency range. However, the rate of increment is different for different audio frequencies. The observed phenomenon can be explained by the connection between the thermal movement of molecules, which make up the amorphous structural material, and dipolar polarisation. Because there is a lack of the necessary thermal energy to offset molecular interaction, dipoles are primarily frozen at lower temperatures. Nonetheless, the thermal energy of dipoles rises together with the system temperature. This facilitates the dipoles to orient themselves by lessening the impact of molecular interaction. In turn, this raises the dielectric constant and polarisation overall. Numerous research teams have documented this behavior in their articles.^30–33^ It can also be noticed that the value of *ε*′ increases with silver halide doping. A dielectric dispersion has been noticed at room temperature. The plots in Fig. 8 depict the correlation between the dielectric constant and frequency for various multicomponent chalcogenide alloys that have been prepared. It is noticeable that as the frequency increases at various temperatures, the *ε*′ shows a downward trend. Dimerization of dipolar polarization is responsible for the observed decrease in *ε*′ values across all compositions with increasing frequency of the applied external electric field. This occurs when the dipoles' oscillations start to lag behind the fields because they can no longer rotate swiftly enough. The material's dipoles are completely unable to keep up with the external field as the frequency increases. This causes the dipolar polarisation to rapidly diminish, which in turn causes the dielectric permittivity value to decrease. The dielectric loss variation of the current samples at various temperatures for constant audio frequencies is depicted in Fig. 9 through graphs. All of these samples experience an increase in dielectric loss with temperature; however, the rate of increase varies depending on the audio frequency. We observe that the *ε*′′ of silver halide doped samples increased as compared to the parent elemental selenium. However, the graph also shows that Se_95_(AgI)_5_ has a minimum increase in dielectric loss. This behavior could be the result of alloy structural orientations changing with temperature, which would reduce the amount of energy dissipated through the material. It is noteworthy that the dielectric loss behavior in these glass-ceramic alloys exhibits complexity and depends on various parameters, such as composition, and external factors (*i.e.*, temperature, and applied frequency). Hence, a comprehensive analysis is necessary to comprehend the fundamental mechanisms that control the *ε*′′ in these materials. The decrease in the number of defect states in the band gap close to the Fermi level is responsible for the decrease in dielectric loss as the silver halides are incorporated.^34–36^

**Fig. 10:**
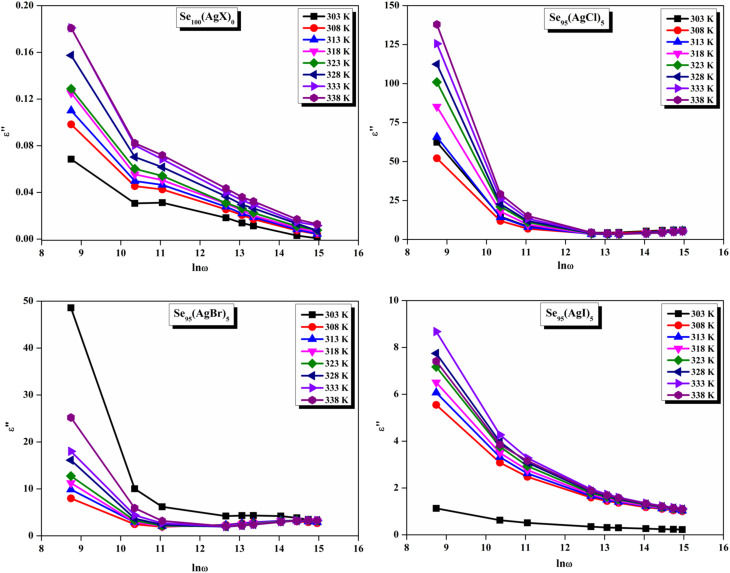
Frequency dependence of dielectric loss for the present samples.

In other words, this reduction in dielectric loss is caused by a decrease in the number of charge carriers hopping within the defect states. Similar results were reported by other research group.^37^ As illustrated in Fig. 10, on the other hand, a drop in the value of *ε*′′ is observed to occur in tandem with an increase in the external field's frequency.

The CBH model describes the conduction mechanism in such substances, which further helps to explain the variation in dielectric loss with frequency.^20,29,38^ According to this paradigm, charge carriers bounce from a donor state to an acceptor state, allowing them to travel between localized sites. When an electron and a hole recombine, they form donor and acceptor sites. Each resulting pair of these sites constitutes a dipole, which exhibits susceptibility to frequency modulation by an external electric field. The energy of the barrier potential that the charge carriers must cross determines the relaxation time of each dipole. Due to the coulombic contact between adjacent sites that form a dipole, this potential barrier is created. This model states that conduction, dipole, and vibration losses are the three distinct mechanisms that give rise to dielectric relaxation.^39^

A close inspection of Fig. 7–10 reveals that the dielectric loss is minimal at all temperatures/frequencies in the case of the doping of AgI as compared to AgCl and AgBr. Numerous studies have elucidated the impact of AgI incorporation on augmenting the optical and electrical properties of various chalcogenide glasses.^40–42^ The Ag^+^ ionic mobility and the subsequent relaxation process account for the majority of the measured overall dielectric response. The remainder, or the localized defect relaxation, is most likely brought about by the host matrix's polarizability, which includes a highly polarizable I^−^ anionic contribution that gets stronger when silver iodide is added to the structure as compared to silver chloride and silver bromide. This probably happened due to the higher polarizability of iodine than that of chlorine and bromine.

**Fig. 11:**
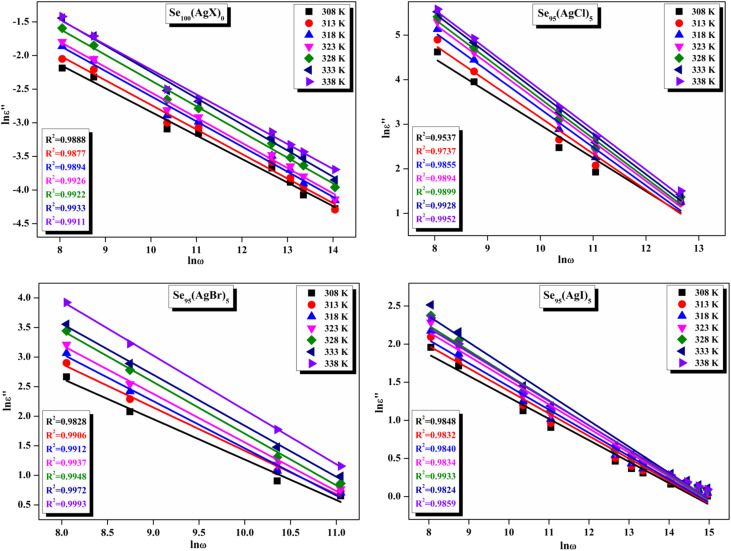
The linear-fitted graph between the natural logarithm of dielectric loss (ln *ε*′′) and the natural logarithm of angular frequency (ln *ω*) for the current samples.

**Fig. 12:**
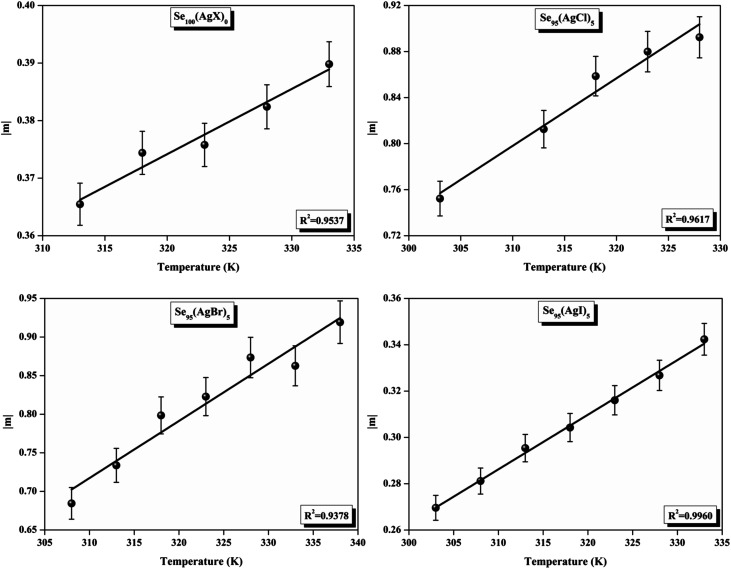
Linear fitted plots of frequency exponent m against temperature *T* (K) for the present samples.

**Fig. 13:**
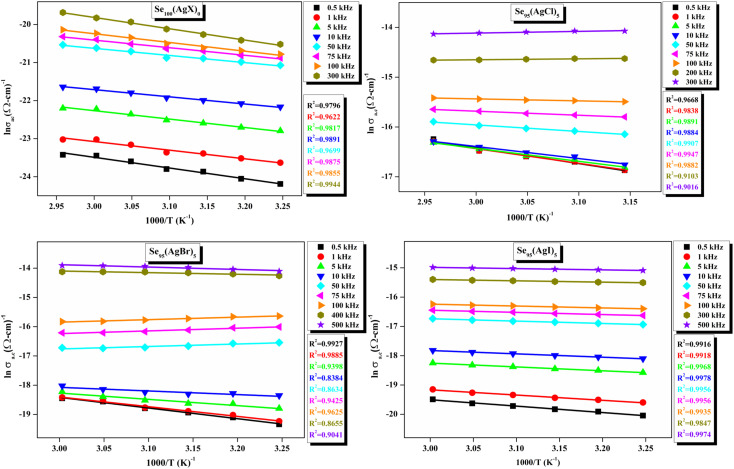
Linear-fitted Arrhenius temperature dependency of ac conductivity (*σ*_ac_) at various audio frequencies.

Fig. 11 presents straight-line fit plots of ln *ε*′′ *versus* ln *ω* for the present variety of amorphous alloys. Since all of the plots in this figure are straight lines with strong correlation coefficients, it sounds likely that a power law may adequately characterize the correlation between ln*ε*′′ and ln*ω*.^24^ Using eqn (2) and the tangent of these straight lines, the values of the power law exponent (*m*) have been determined. In the silver iodide doped sample, at the higher frequency, there is the conversion of ln*ε*′′ values. The power law relationship between ln*ε*′′ and ln*ω* can be used to understand how the dielectric characteristics of various substances behave over a range of frequencies because it is an inherent feature of many of them. These findings suggest that the doping of silver halides significantly influences the temperature-dependent variation of dielectric parameters. The change in *m* against temperature is shown in Fig. 12. The linear dependence of these graphs on temperature agrees with relation (4), supporting the relevance of Guintini's hypothesis.^18^ The behavior of the dielectric characteristics of the amorphous selenium silver halide alloys is adequately explained by this theory. A popular idea to describe how materials behave dielectrically at different frequencies is called Guintini's theory. It suggests that the motion of charged particles in an electric field is connected to a material's polarisation response. In essence, these plots support the relevance of Guintini's theory. Therefore, the linear dependency of the *m*-scale magnitudes on temperature follows the relation outlined by eqn (4), aiding in comprehending the dielectric behavior of sample alloys. The temperature dependency of AC conductivity has been evaluated for all the prepared samples. Fig. 13 depicts the variation of ln*σ*_ac_ against 1000/*T* for parent elemental selenium and silver halide doped chalcogenide samples. The plot of ln*σ*_ac_ against 1000/*T* for all samples form a straight line, suggesting a thermally activated process for AC conduction. The diagrams indicate that the AC conductivity's reliance on temperature adheres to the Arrhenius equation:15
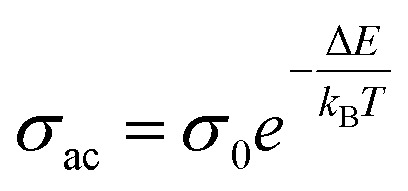


**Fig. 14:**
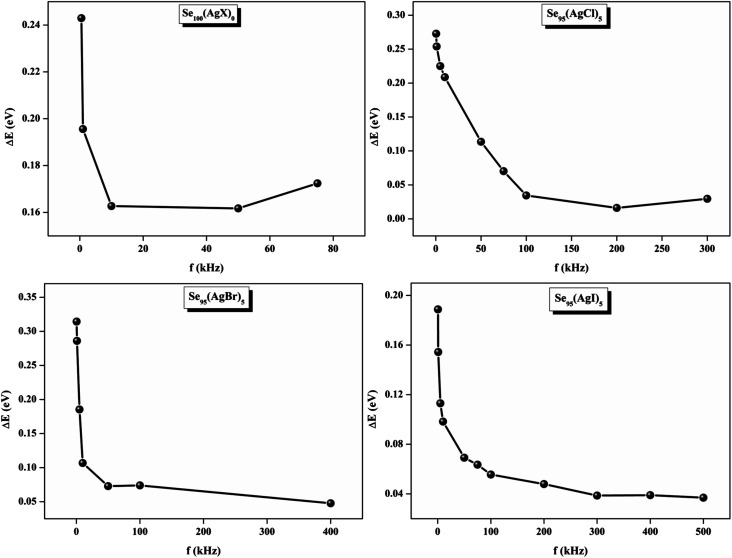
Frequency dependence of activation energy Δ*E*_ac_ for all four samples.

**Fig. 15:**
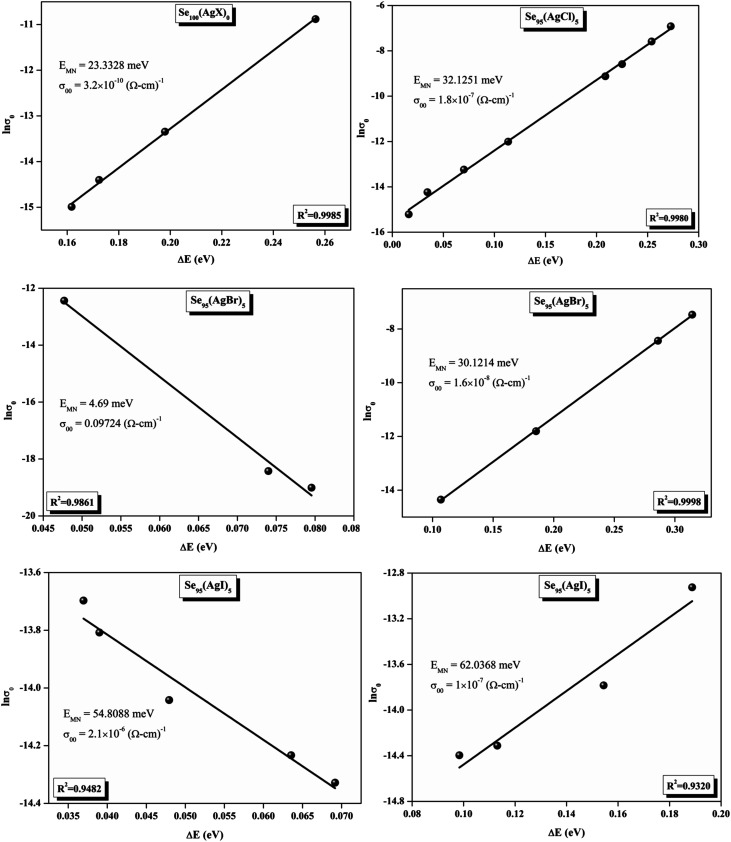
Meyer–Neldel plots for AC conduction in as-prepared silver halide chalcogenide samples.

At higher frequencies, it can be observed that in the silver halide-doped samples conductivity became almost independent of the temperature. The correlation between electrical conductivity and temperature suggests a conduction behavior where electrons hop, activated by heat.^43^Fig. 14 presents the frequency-dependent Δ*E*_ac_ for all the prepared materials. It shows that when frequency rises, activation energy decreases. While the observed pattern is nearly constant across all compositions, the values vary according to frequency. The activation energy, which represents the energy needed for charge carriers to move through the material, gets smaller as frequency increases. This implies that charge carriers will travel more easily at higher frequencies. This phenomenon is linked to localized states that trap and release charge carriers. At lower frequencies, these states can impede carrier motion and increase activation energy due to prolonged interaction time. Lower activation energy and freer carrier movement are made possible at higher frequencies by shorter contact times. Beyond a particular frequency, the activation energy stabilizes, suggesting that the higher frequencies have less of an impact from localized states. This behavior is consistent with the frequency-dependent dielectric constant and is typical of disordered materials. Essentially, the patterns seen in Fig. 15 and other compositions emphasize that activation energy possesses a frequency dependence that is a crucial characteristic of disordered materials.^29,44^ The experiments propose a correlation between the activation energy of thermally activated AC conduction and its corresponding pre-factors:16
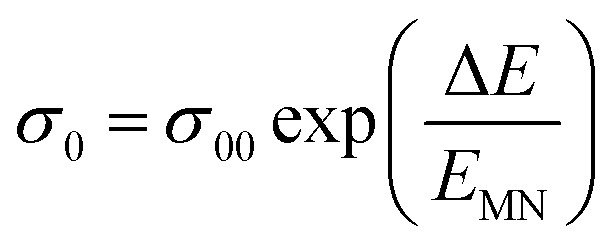


**Fig. 16:**
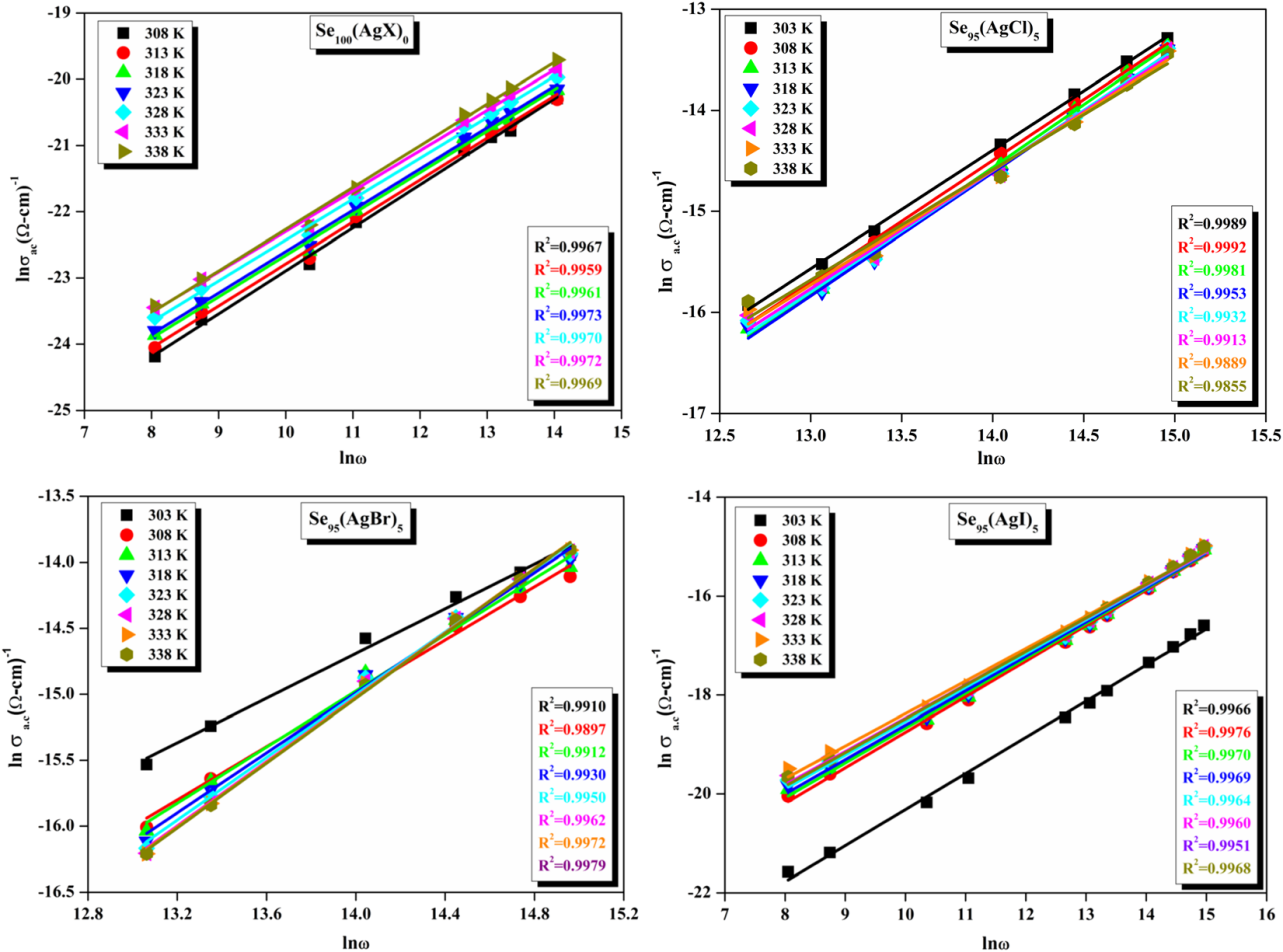
Frequency dependence of AC conductivity for all samples at different temperatures.

**Fig. 17:**
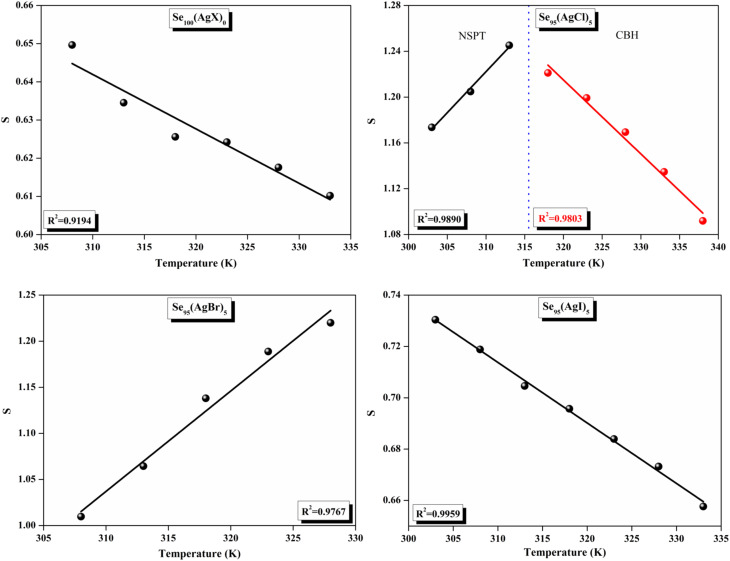
Temperature dependence of the frequency exponent (*S*).

**Fig. 18:**
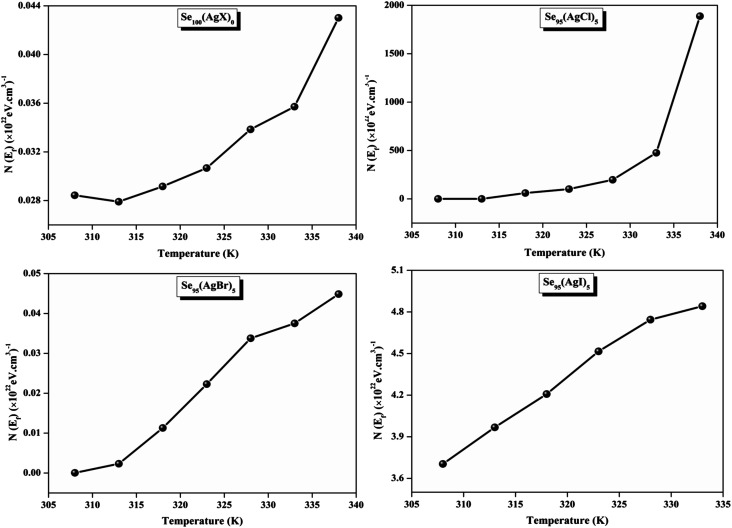
Variation of the density of defect states with increasing temperature.

The relationship between the activation energy (Δ*E*) and AC conductivity, which is known as the Meyer–Neldel empirical rule,^45^ and the pre-factor *σ*_0_ is depicted in eqn (16). The term *E*_MN_ is referred to as Meyer–Neldel energy. The Meyer–Neldel Rule (MNR), an established principle in the domain of materials science, finds application across diverse substances, notably chalcogenide glasses,^46^ and various semiconductor classifications. Through the utilization of the gradients inherent in these linear representations, the determination of the activation energy values (*E*_MN_) was achieved *via* linear regression analysis of the plotted data, as demonstrated in Fig. 15. Based on the graphs, it's observed that the value of *E*_MN_ shows significant changes after silver halide doping in g-Se. We observed that the parent and sample doped with silver chloride followed the MNR rule, whereas the remaining samples followed MNR and reverse MNR in different regions of activation energy corresponding to AC conductivity. A rise in activation energy in the standard MNR is correlated with a boost in the pre-exponential factor. On the other hand, a drop in the pre-exponential component would be associated with an upsurge in activation energy in a reverse MNR trend. The detection of both MNR and reverse MNR trends in the samples doped with silver bromide and silver iodide may be the result of microstructure variations and accompanying shifts in the effective density of state distributions. However, interpreting these trends requires a deep understanding of the material's properties and the specific processes contributing to its behavior. Therefore, further investigation would be needed to determine the exact cause of these trends. Fig. 16 illustrates how the AC conductivity varies with frequency. The straight lines fitting that the least-squares fitting method produced is depicted in the graph by solid lines. As inferred from Fig. 16, within a temperature range of 303–338 K, the AC conductivity shows an upward trend with the rise in frequency. In disordered materials, there are often areas known as localized states. These are regions where, due to the disorder in the material's structure, charge carriers can become temporarily trapped. When an alternating current (AC) is applied to the material, it causes these charge carriers (D^+^ and D^−^) to hop in and out of these localized states. This hopping process contributes to the overall conductivity of the material. As the frequency of the *σ*_ac_ increases, each cycle of the current is shorter. Charge carriers will therefore have fewer opportunities to get stuck in these localised states. Rather, they are more probable to aid in the material's ability to conduct current. Consequently, we see a boost in AC conductivity at higher frequencies. This is because more charge carriers are involved in conducting current rather than being trapped in localized states. This phenomenon is common in disordered materials and is a key factor in understanding their electrical properties. It is also important in the design and application of devices that use these materials. The frequency exponent *s* described in eqn (5) is calculated by slopes of straight lines. Fig. 17 displays the temperature variation of *s* for each sample. Fig. 17 makes it evident that for both the parent Se and the Se_95_(AgI)_5_ sample, the frequency exponents fall as the temperature rises, suggesting that the CBH model may be used to describe the conducting mechanisms in both cases.^20^ Hopping conduction phenomena are typically characterized by the value of the exponent *s*, which is in the range of 0 and 1.^47,48^ The energy barriers dividing localized states get thinner as temperature increases. As a result, conductivity may increase and the frequency exponent “*s*” may decrease. On the other hand, for Se_95_(AgCl)_5_ and Se_95_(AgBr)_5_ story looks different. For the Se_95_(AgCl)_5_ sample, the value of *s* increases first in the temperature range from 303 K to 313 K, and then *s* decreases in the temperature range 318 K to 338 K. In the first region, the NSPT model is an appropriate model for the conductivity whereas the modified CBH model best explains the conductivity in the second region. For the Se_95_(AgBr)_5_ sample, there is a rise in the value of *s* throughout the temperature range. The NSPT model could account for the conduction mechanism.^49^ In addition, we computed the density of defect states using the models that were used. Plots from Fig. 18 show that the density of localized states *N*(*E*_f_) in all samples increases with temperature. The value of *N*(*E*_f_) increases insignificantly, moderately, and drastically when we add AgBr, AgI, and AgCl in parent selenium glass respectively.^34–36^Table 1 contains the resulting values of hopping length and density of localized states at temperature 308 K.

**Table 1:** The density of charged defect states and the values of polaron hopping distance of Se_100−*y*_(AgX)_*y*_ (where X = Cl, Br, and I; *y* = 0, and 5) alloys at frequency 1 kHz and temperature 308 K

Sample	*R*_*ω*_ (Å)	*N*(*E*_f_) (eV cm^3^)^−1^
Se_100_(AgX)_0_	14.9	2.8 × 10^20^
Se_95_(AgCl)_5_	97.6	5.4 × 10^23^
Se_95_(AgBr)_5_	2049.2	4.0 × 10^17^
Se_95_(AgI)_5_	5.8	3.7 × 10^23^

To maximize the dielectric characteristics of multi-component ChGs including silver, such as low dielectric loss and a high dielectric constant, researchers have spent the last thirty-four years examining the dielectric behavior of these compounds with binary, ternary, and quaternary compositions. We have contrasted the *ε*′ and *ε*′′ values of the current samples with those of such multi-component ChGs.^2,33,50–55^Table 2 tabulates the comparative analysis of these data. This table reveals that, in comparison to previous silver-containing multi-component ChGs published in the literature, the current system incorporating silver halide optimizes the dielectric properties better. Notably, the silver iodide-doped sample exhibits the best results.

**Table 2:** Comparisons of the dielectric constant and loss at room temperature (305 to 310 K) and 1 kHz frequency for silver-halide containing selenium glass-ceramics in the present study with the available data of silver containing chalcogenide glasses in the literature of the last decade

S. No.	Composition	ε′	ε′′	Reference
1	Se_70_Te_28_Ag_2_	742	936	50
	Se_70_Te_26_Ag_4_	806	810	
	Se_70_Te_24_Ag_6_	704	1060	
	Se_70_Te_22_Ag_8_	134	242	
	Se_70_Te_20_Ag_10_	1085	325	
3	Se_76_Te_20_Sn_2_Ag_2_	62.2	895.4	51
	Se_74_Te_20_Sn_2_Ag_4_	91.9	1746.9	
	Se_72_Te_20_Sn_2_Ag_6_	47.6	185.7	
4	Se_98_Ag_2_	6.85	0.9	52
	Se_96_Ag_2_In_2_	12.8	1.7	
	Se_94_Ag_2_In_4_	9.5	0.66	
	Se_92_Ag_2_In_6_	6.35	0.27	
5	Se_75_Ge_20_Ag_5_	21.5	2.5	53
6	Se_65_Te_20_Ag_15_	30.5	—	54
7	Se_92_Te_4_Ag_4_	45	25	55
	Se_88_Te_4_Ag_8_	23	14	
	Se_84_Te_4_Ag_12_	20	10	
	Se_80_Te_4_Ag_16_	17	8	
10	Se_100_(AgX)_0_	8.47	5.6	Present work
	Se_95_(AgCl)_5_	25.9	31.9	
	Se_95_(AgBr)_5_	16.21	7.9	
	Se_95_(AgI)_5_	17.58	5.5	

## 6. Conclusions

This study investigates the AC conductivity and dielectric relaxation processes in newly developed chalcogenide semiconductors doped with silver halides (AgX, where X = Cl, Br, I) over a wide range of frequencies and temperatures. The observed changes in the dielectric constant (*ε*′) and dielectric loss (*ε*′′) are attributed to dipolar and electronic polarization, which are influenced by structural modifications within the material. Doping with AgX significantly enhances the dielectric properties, with an increase in defect state density[*N*(*E*_f_)] explaining the variations in *ε*′ and *ε*′′. The samples exhibit AC conductivity dispersion consistent with Jonscher's universal power law. The Meyer–Neldel (MN) rule effectively explains the AC conductivity data. The results indicate that thermally stable silver halide-doped glass ceramics can be produced, with silver iodide providing the optimal balance of dielectric constant and loss, followed by silver chloride and silver bromide. These AgX-doped selenium glass ceramics demonstrate superior dielectric performance compared to other silver-containing chalcogenide glasses studied by different research groups. The incorporation of AgX enhances the electrical properties of chalcogenide materials, making them suitable for various solid state device applications.

## Author contributions

Anil Kumar, and Vishnu Saraswat: plotting graphs, wrote-original draft; A. Dahshan and H. I. Elsaeedy: wrote a revised version of the manuscript; N. Mehta: formal analysis, conceptualization, wrote-final draft.

## Conflicts of interest

There are no conflicts to declare.
